# Unleashing the Power of Artificial Intelligence in Materials Design

**DOI:** 10.3390/ma16175927

**Published:** 2023-08-30

**Authors:** Silvia Badini, Stefano Regondi, Raffaele Pugliese

**Affiliations:** NeMO Lab, ASST GOM Niguarda Cà Granda Hospital, 20162 Milan, Italy; silvia.badini@nemolab.it (S.B.); stefano.regondi@nemolab.it (S.R.)

**Keywords:** artificial intelligence, machine learning, materials design, materials informatics, materials properties prediction

## Abstract

The integration of artificial intelligence (AI) algorithms in materials design is revolutionizing the field of materials engineering thanks to their power to predict material properties, design de novo materials with enhanced features, and discover new mechanisms beyond intuition. In addition, they can be used to infer complex design principles and identify high-quality candidates more rapidly than trial-and-error experimentation. From this perspective, herein we describe how these tools can enable the acceleration and enrichment of each stage of the discovery cycle of novel materials with optimized properties. We begin by outlining the state-of-the-art AI models in materials design, including machine learning (ML), deep learning, and materials informatics tools. These methodologies enable the extraction of meaningful information from vast amounts of data, enabling researchers to uncover complex correlations and patterns within material properties, structures, and compositions. Next, a comprehensive overview of AI-driven materials design is provided and its potential future prospects are highlighted. By leveraging such AI algorithms, researchers can efficiently search and analyze databases containing a wide range of material properties, enabling the identification of promising candidates for specific applications. This capability has profound implications across various industries, from drug development to energy storage, where materials performance is crucial. Ultimately, AI-based approaches are poised to revolutionize our understanding and design of materials, ushering in a new era of accelerated innovation and advancement.

## 1. Introduction

The advancement of human society greatly relies on materials design, which holds significant influence across a wide range of applications, ranging from civil engineering to regenerative medicine [1].

Historically, the discovery and design of new materials relied heavily on chance and the “trial-and-error method” based on experimentation guided by experience, often through serendipitous discovery [2,3]. A typical materials discovery effort can be divided into a series of phases [4]. Firstly, researchers identify a specific research question or objective. Then, they gather relevant existing data to inform their investigation. Based on this information, a hypothesis is formulated, leading to the subsequent phase of experimentation and testing. Through this iterative process, new knowledge is generated, giving rise to further hypotheses. Despite the apparent simplicity of this framework, numerous bottlenecks impede its smooth execution, resulting in the slow and time-consuming nature of materials design and discovery. Indeed, it can take several years, if not decades, for initial exploratory work on a novel material concept to reach a stage where it becomes a market-ready product [5].

In particular, one of the most challenging topics in this field is searching for effective methods to find and design new materials with optimal mechanical, thermal, biological, and chemical properties, which ensures that the materials can consistently work as designed without failures. 

The rapid advancements in artificial intelligence (AI) and machine learning (ML) hold immense potential for revolutionizing and expediting the arduous and costly process of materials development. In recent decades, AI and ML have ushered in a new era for materials science by leveraging computer algorithms to aid in exploration, understanding, experimentation, modeling, and simulation [4,6]. Working alongside human creativity and ingenuity, these algorithms contribute to the discovery and refinement of novel materials for future technologies. 

According to Philip Ball [2], computer algorithms have now developed a form of intuition by identifying patterns and regularities within existing knowledge, mirroring the processes used by scientists. By learning from experience, these algorithms can assist researchers in selecting and designing experiments, analyzing results, and extracting generalized knowledge. This approach, which involves digesting and generalizing existing knowledge to find innovative solutions, has found applications across various domains where copious amounts of data surpass human assimilation capabilities, including genomics, drug design, and financial market analysis. Consequently, it is increasingly probable that similar methodologies will tackle outstanding challenges in materials design, such as mechanical materials [7], bioinspired materials emulating the multifunctionality of biological counterparts [8], or self-healing architectured metamaterials [9]. For instance, Lu et al. [10] presented a graph-focused deep learning technique to capture the intricate design nuances found in spider web architectures. This technique was harnessed not only to understand these complexities but also to facilitate the generation of a wide spectrum of novel bioinspired structural designs. By doing so, the authors have established a seminal framework for the generation of spider webs while delving into the realm of bioinspired design guided by rigorous principles. Beyond its immediate application to spider web emulation, this method boasts versatility in tackling a variety of heterogeneous hierarchical structures. Encompassing a broad spectrum of architected materials, it stands poised to illuminate fundamental biological insights and to address a diverse array of design prospects. Through the lens of generative AI for materials applications, this approach emerges as a powerful toolset, bridging the gap between theoretical exploration and practical design actualization.

Bo Ni et al. [11] introduced an innovative deep learning framework centered on diffusion models, skillfully tailored to facilitate the efficient design of materials with precise molecular control. The study’s focal point was the creation of de novo protein sequences, an exemplification of a major engineering endeavor that holds potential for nanotechnology. Leveraging the rich potential of proteins, which draw inspiration from nature’s toolkit to construct a diverse array of biotic, abiotic, and hybrid materials, the researchers have unveiled a potent avenue to meet this challenge. However, it is exceedingly difficult to invent new proteins that go beyond evolutionarily obtained solutions. The authors showed that the sequences generated through this approach exhibit a remarkable novelty that transcends established natural variants. By proficiently crafting an assortment of inventive sequences, each endowed with the desired structural attributes, the framework offers expedited strategies for a targeted and pioneering pursuit of de novo protein design. This, in turn, engenders the discovery of exceptional protein materials suitable for a wide array of biological and engineering applications. Importantly, the implications of this model extend beyond its current manifestation, suggesting its potential for future pursuits aimed at diverse design objectives.

Thus, AI has the potential to usher in a novel scientific paradigm, enhancing, streamlining, and guiding the acquisition of new knowledge about the vast material universe while mitigating or eliminating research bottlenecks [12]. It promises to provide a transformative approach, propelling the process of materials discovery towards unprecedented levels of efficiency and effectiveness. In the meantime, different research articles on specific designed materials using AI, involving energy materials [13], composites [14], polymers [15], bioinspired materials [16], and additively manufactured materials [17], are coming out.

In light of this, a comprehensive overview of the research efforts focused on materials design utilizing AI and ML algorithms is provided here. Section 2 addresses the state-of-the-art advancements in AI models within the field of materials design. This encompasses supervised learning, unsupervised learning, reinforcement learning, and the utilization of material informatics tools.

**Figure 1:**
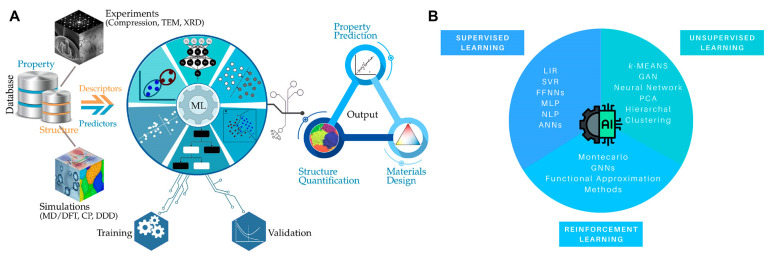
(**A**) A schematic representation of the materials design workflow using AI, consisting of three essential elements: a material dataset, machine learning models capable of learning and interpreting representations for specific tasks using the provided dataset, and output that yields optimized and enhanced material properties for the creation of advanced materials. Reproduced with permission from Ref. [22]. CC BY 4.0 (**B**) An overview of machine learning methodologies highlighting the three primary categories: supervised learning, unsupervised learning, and reinforcement learning.

These methodologies empower researchers to extract meaningful insights from massive datasets, unraveling intricate correlations and patterns embedded within material properties, structures, and compositions. It is crucial to bear in mind the three key components that constitute a typical workflow, as illustrated in Figure 1A, when combining AI and materials research: (1) a material dataset sourced from the literature, existing databases, or generated through experiments and simulations; (2) an ML model equipped with the capability to learn and interpret representations for specific tasks; and (3) an output that provides solved data, facilitating the discovery of new materials designs with optimized and enhanced features.

Subsequently, we delve into a comprehensive discussion on the application of ML methods in addressing various material design challenges. In Section 3 we overview the field of biologically inspired materials, while prediction of material mechanical behavior is analyzed in Section 4. Lastly, the complexities associated with soft materials and compositionally complex metamaterials are discussed in Section 5 to clarify their advantages and highlight their potential. In Section 6, conclusions are drawn.

Through this review, we aspire to provide a comprehensive understanding of the current state of the art, shedding light on the immense potential and breakthroughs that AI and ML offer in materials design.

Although we are still in the early stages of this transformative journey, as Riley aptly cautioned [2], envisioning a future where all materials scientists possess AI-based co-pilots, akin to our everyday companions such as Google Assistant or Alexa, opens up a realm of vast possibilities. With AI tools by their side, the capabilities and potential for materials scientists expand significantly, promising a future where innovation and progress in the field can reach unprecedented heights. 

## 2. AI Algorithms and Material Informatics Tools

### 2.1. AI Algorithms and ML Models

AI and ML have revolutionized the way we approach problem-solving and decision-making [18,19,20]. ML, in particular, is a field that focuses on the development and deployment of AI algorithms capable of analyzing data and its properties to determine actions without explicit programming [21]. 

Unlike traditional programming, ML algorithms leverage statistical tools to process data and learn from it, allowing them to improve and adapt dynamically as more data becomes available. This concept of “learning” forms the foundation of ML, enabling algorithms to make predictions, recognize patterns, and make informed decisions.

ML algorithms can be broadly categorized into three main types, each serving different purposes: supervised learning, unsupervised learning, and reinforcement learning. These diverse categories of ML algorithms provide a powerful toolkit for solving complex problems, optimizing processes, and extracting valuable insights from data. As illustrated in Figure 1B, they form a comprehensive framework that enables AI systems to analyze and interpret data, facilitating intelligent decision-making and automation across various domains.

Supervised learning involves training algorithms using labeled data, where inputs and desired outputs are provided to teach the algorithm how to make accurate predictions. Hence, this learning process is based on comparing the calculated output and predicted output, that is, learning refers to computing the error and adjusting the error for achieving the expected output. Examples of such algorithms include linear regression (LIR) [23], support vector regression (SVR) [24], feedforward neural networks (FFNNs) [25], and convolutional neural networks (CNNs) [26]. 

In addition to classical supervised ML algorithms such as LIR, SVR, or random forests (RFs), which are useful for predicting mechanical features of materials [7], researchers have developed artificial neural networks (ANNs) inspired by the interconnected neurons in the human brain to delve into deep data mining [27]. Among these networks, FFNNs, or multilayer perceptrons (MLPs), have emerged as quintessential and relatively simple models. FFNNs are extensively used in ML and deep learning, featuring multiple layers of interconnected nodes or neurons arranged sequentially. These networks facilitate data flow in a unidirectional manner, from the input layer to the output layer, without any loops or feedback connections.

Each layer, comprising multiple neurons, calculates outputs for the subsequent layer based on inputs received from the preceding layer. The weights or trainable parameters associated with each neuron are optimized to minimize the loss function, allowing the FFNN to learn complex patterns and relationships in the data.

In the field of materials design, FFNNs can be effectively employed in various ways [28,29,30]. For instance, by providing the composition, processing conditions, and microstructure of a material as inputs, an FFNN can learn the intricate relationship between these factors and properties such as mechanical strength, thermal conductivity, or electrical resistivity. This enables the optimization of material compositions and processing parameters to achieve desired properties. Furthermore, FFNNs can be combined with optimization algorithms to explore and optimize material designs [31,32]. By treating the FFNN as a surrogate model that approximates the relationship between input variables (e.g., material composition, processing conditions) and desired performance metrics, optimization algorithms can efficiently search the design space to identify optimal material configurations. This application proves particularly valuable when seeking materials with specific properties or performance targets. 

However, it is important to note that successful utilization of FFNNs in materials design relies on the availability of high-quality training data, careful feature selection, and a thorough understanding of the model’s limitations and assumptions. Additionally, domain expertise and experimental validation remain crucial in interpreting and verifying the FFNN’s predictions and recommendations.

Besides FFNNs, CNN architectures are gaining widespread attention due to their applications in computer vision and natural language processing (NLP) [33]. They serve as a specialized type of ANN, specifically designed for analyzing visual data such as images or videos. CNNs possess the ability to automatically learn hierarchical representations of visual patterns and features directly from raw input data. The fundamental operation in CNNs is convolution, which preserves the spatial relationship between pixels. It involves multiplying the image matrix with a filter matrix, where the filter contains trainable weights that are optimized during the training process for effective feature extraction. By employing various filters, CNNs can perform distinct operations such as edge detection on an image. Through the stacking of convolutional layers, simple features gradually combine to form more complex and comprehensive ones.

CNNs have revolutionized computer vision tasks, encompassing image classification, object detection, segmentation, and more [33]. Their hierarchical structure, parameter sharing, and spatial invariance properties contribute to their efficacy in learning and extracting meaningful features from visual data. Consequently, CNNs have found widespread adoption in numerous domains, including medical imaging, facial recognition, and image-based recommender systems [34,35].

In the field of material design problems, CNNs hold great potential. With their capability to capture features at different hierarchical levels, CNNs are well-suited for describing the properties of materials, which inherently possess hierarchical structures, particularly in the case of biomaterials [36].

Overall, CNNs offer a powerful toolset for extracting relevant information from visual data, enabling breakthroughs in a wide range of applications and fostering advancements in fields such as materials engineering and design.

Unsupervised learning, on the other hand, deals with unlabeled data, where the algorithm identifies patterns and structures within the data without explicit guidance. Intriguing and successful categories of unsupervised architectures are generative adversarial networks (GANs), which consist of two neural networks, the generator and the discriminator [37]. GANs are designed to learn and generate synthetic data that resembles a target dataset, without the need for explicit labels or supervision. The generator network is responsible for generating synthetic data samples. It takes as input random noise or a latent vector and produces data that resembles the target dataset. The generator network usually consists of one or more layers of neural nodes, often using deconvolutional layers to up-sample the noise into a larger output. The discriminator network acts as a binary classifier, distinguishing between real data samples from the target dataset and synthetic data samples generated by the generator network. The discriminator network aims to correctly classify whether a given sample is real or fake. It is trained with labeled data, where real samples are labeled as “real” and synthetic samples as “fake”. GANs have gained significant attention in the field of ML and have shown impressive capabilities in generating realistic and diverse data, including images, text, and audio [38]. They have also shown potential in unsupervised learning tasks, where the generated data can be used for downstream tasks such as clustering, representation learning, and semi-supervised learning. It’s important to note that GANs require careful tuning, hyperparameter selection, and large amounts of training data to achieve optimal performance. Additionally, evaluation metrics for GANs are an ongoing area of research, as assessing the quality and diversity of generated samples can be subjective.

In a notable study, Mao et al. [39] introduced a GAN-based approach for designing complex architectured materials with extraordinary properties, such as materials achieving the Hashin-Shtrikman upper bounds on isotropic elasticity. This method involves training neural networks using simulation data from millions of randomly generated architectured materials categorized into different crystallographic symmetries. The advantage of this approach lies in its ability to provide an experience-free and systematic framework that does not require prior knowledge and can be readily applied in diverse applications.

The significance of this methodology extends beyond the design of metamaterials. By leveraging simulation data and ML, it offers a novel and promising avenue for addressing various inverse design problems in materials and structures. This approach opens up exciting possibilities for tackling complex design challenges and exploring new frontiers in materials science and engineering. The work by Mao and colleagues not only contributes a practical and systematic method for designing materials with desired properties but also highlights the potential of combining simulation data and ML in materials research. By harnessing the power of generative adversarial networks, this approach enables the exploration of vast design spaces and paves the way for innovative advancements in materials design and engineering.

Lastly, reinforcement learning focuses on training algorithms through interactions with an environment, where the algorithms learn by receiving feedback and rewards based on their actions. Among this class of ML algorithms, AlphaFold must surely be mentioned. It is a reinforcement learning-based AI system developed by DeepMind that has demonstrated exceptional capabilities in predicting protein structures [40]. It utilizes deep learning algorithms to accurately predict the 3D structure of proteins, which is a challenging and crucial task in the field of biochemistry and molecular biology. While initially focused on protein folding, the underlying principles and techniques of AlphaFold have the potential for broader applications, including materials problems such as interactive materials design. 

Besides this outstanding example, graph neural networks (GNNs) belong to this class of ML algorithms. GNNs are a type of ANN designed to process and analyze graph-structured data [41]. Graphs are mathematical structures that consist of nodes connected by edges. GNNs are specifically developed to capture and model the relationships and dependencies between nodes in a graph. GNNs operate on graph-structured data, which can represent various real-world systems and relationships. Each node in the graph represents an entity, while the edges denote the connections or relationships between entities. Using GNNs, Guo and Buehler [42] have developed an approach to design architected materials. The GNN model is integrated with a design algorithm to engineer the topological structures of the architected materials. The authors reported that such sensing method is applicable to design problems of truss-like structures under complex loading condition in additive manufacturing (e.g., 3D printing), architectural design, and civil infrastructure applications, and has the potential to be closely integrated with IoT methods and autonomous sensing and actuation approaches.

ML algorithms used in materials design, along with example applications, are summarized in Table 1.

### 2.2. Materials Informatics Methodologies

Besides the development of AI and ML models for processing and analyzing massive amounts of data, discovering patterns, and making predictions, materials informatics (MI)—a multidisciplinary field that act as a junction between materials science, data science, and AI—has the capabilities to unlock the potential of vast material database management, thus accelerating materials design and development. By integrating MI tools with AI and ML algorithms (namely Hybrid AI), researchers can draw on the multitude of available data and extract valuable insights that were previously unreachable, revolutionizing the way of engineering materials in several fields of application [51,52,53].

The framework of MI mainly consists of three parts: (1) data acquisition, (2) data representation, and (3) data mining (or data analysis) [54].

Data acquisition involves obtaining physical and structural properties through simulations or experiments. Data representation focuses on selecting descriptors that capture the essential characteristics of materials within a dataset. Lastly, data mining aims to identify relationships between structural information and desired material properties [54].

MI methodologies are various and tailored to address specific design challenges. The choice of methodology depends on the nature of the problem and the objectives of the research. Among such methods, the most used for materials design are integrating data modalities, physical-based deep learning, materiomics, and computer vision methodologies. An overview of the main methodologies employed in MI along with their key features is reported in Table 2. 

Integrating data modalities are powerful data acquisition tools. Transformer models, in particular, provide a robust framework for combining multiple sources of multimedia data, such as text, images, videos, and graphs, thereby expanding and enhancing datasets. This integration capability has empowered researchers, exemplified by the work of Hsu et al. [55], to design sustainable materials derived from biocompatible resources with greater efficacy. By leveraging the rich information contained in various data formats, transformer models facilitate the optimization of mechanical properties, starting from the microstructure level [56]. This integration of data modalities unleashes the potential for comprehensive materials analysis and design, opening up new avenues for advancing the field of sustainable materials. To permeate collected data with significance, physical principles are integrated into deep learning techniques, resulting in efficient simulations. A deep learning method has been used to predict high-fidelity and high-resolution images for stress fields near cracks considering material microstructures [57]. Additionally, physics-informed neural networks have been utilized to derive data-driven solutions for nonlinear partial differential equations, smoothing the modeling of dynamic problems [58]. Furthermore, as reported by Lai and co-workers [59], a data-driven regression model demonstrated the correlation between the crystalline structure and luminescence characteristics of Europium-doped phosphors, enabling the prediction of emission wavelengths.

To achieve superior material properties starting from the design phase, it is fundamental to understand the intricate interplay between the physical, chemical, and topological properties of matter. In this content, materiomics employs analytically driven, simulation-driven, and data-driven procedures to predict complex behaviors by breaking down materials into their hierarchical building blocks. This approach is particularly valuable for bioinspired material design, as it considers relevant scales and draws inspiration from the well-organized structures found in nature [60]. Furthermore, materiomics offers a promising avenue to ensure the environmental sustainability of manufactured structures, as it considers the life cycle and ecological impact of materials [61]. Moreover, the use of AI enables the resolution of inverse design problems in order to develop material compositions and structures that fulfill a specific set of target requirements. These requirements often involve challenging requirements, such as enhancing mechanical performance or efficiency while simultaneously reducing weight and cost [62,63,64,65].

To enhance the interpretability of results in materials engineering, computer vision methodologies, such as graphic rendering and virtual reality, can be implemented. Indeed, Yang et al. [66] employed an AI-based approach by using molecular dynamics simulations to realize the structure and property quantification of 3D graphene foams with mathematically regulated topologies. In another study the authors, using a limited set of known data and a multiple deep learning architecture, demonstrated the capability to predict missing mechanical information and further analyze intricate 2D and 3D microstructures [67]. Furthermore, the training of specific algorithms can provide extra information on mechanical features that can optimize the material design process and lead scientists to new discoveries [68,69,70].

Another data mining tool is represented by the possibility of transfer learning and fine-tuning the algorithms. This methodology considers the adaptation of pre-existing models to address problems that differ from the original one, thereby altering the characteristics of the input data or the reward value [71]. For instance, Jiang et al. [72] used a transfer learning algorithm that solved dynamic multi-objective optimization problems to generate an effective initial population pool via reusing past experience to speed up the design process. 

Currently, the utilization of large language models (LLMs), such as Chat-GPT, LLaMa, and Bard, is generating significant interest due to the profound impact this technology can have on human life [73,74]; moreover, its application in material analysis can be considered as a valuable instrument for intelligent material design and prototyping [75]. One notable advantage is the ability to fine-tune LLMs for specific tasks using a relatively small amount of labeled data, which can even be extracted from the published literature [76]. This approach eliminates the need to train a new model from scratch. Indeed, the final layers of the neural network can be substituted to adapt the AI parameters, and then the entire model can be trained in significantly less time than training it from scratch would take. These adaptations enable LLMs to be effectively applied in various domains, including dataset mining, molecular modeling, microstructure generation, and material structure extraction [77,78]. 

Lastly, another powerful tool in materials research is the autonomous discovery of materials using AI. By harnessing the capabilities of automated experimentation systems, laboratories empower AI to autonomously explore the extensive design space and make informed decisions about which experiments to conduct. This approach revolutionizes the traditional trial-and-error approach to materials discovery by leveraging AI algorithms and ML techniques. The AI system can analyze vast amounts of data, including experimental results, material properties, and synthesis conditions, to identify patterns, correlations, and novel material candidates [79,80]. Through this autonomous exploration, complex new materials with unprecedented characteristics are unveiled [81]. An interesting development in this field was presented by Nikolaev et al. [82] who established the first autonomous experimentation (AE) system for materials development. Initially, the AE system was trained to grow carbon nanotubes with precise growth rates by applying a six-dimensional processing parameter gained after obtaining a deeper understanding of the underlying phenomena. Through an iterative research process spanning 600 autonomous iterations, the AE system successfully identified the optimal growth conditions to achieve the desired growth rate.

Overall, to further enhance the materials discovery process, the coupling of MI tools with AI algorithms demonstrates vitality. This combination deploys the possibility to renovate and transform research methodologies. By leveraging digital strategies, researchers can overcome traditional challenges encountered in materials design, thus boosting the discovery and development of novel materials.

**Table 2:** Key methodologies in materials informatics.

Materials Informatics (MI) Tools		
Type	Main Features	References
Integrating data modalities	Integration of different multimedia sources into datasets (text, images, videos, and graph data)	[55,56]
Physics-based deep learning	Integration of physics models into deep learning settings	[57,58,59]
Materiomics	Usage of analytically driven, simulation-driven, and data-driven methods to break down materials into their essential building blocks	[60,61]
Inverse design	Solving inverse design problems	[62,63,64,65]
Computer vision methodologies	Combination of graphic rendering, virtual reality, and interpretable machine learning	[66,67]
Transfer learning and Fine-tuning	Adaptation of pre-existing algorithms to a different problem resolution	[71,72]
Large Language Models (LLMs)	Elaboration of various natures of datasets to predict and generate text and other forms of content	[75,76,77,78]
Autonomous materials discovery	Autonomous exploration of design space with self-directed decisions regarding experimentation and tests	[79,80,81,82]

## 3. AI Algorithms in Biologically Inspired Materials

In the field of materials design, researchers have long been captivated by the possibility of obtaining materials with superior properties and multifunctionality. However, progress in the development of completely new materials has been relatively slow and challenging. One key area of interest lies in biological materials, which possess unique features due to their elementary nature which is constituted by simple building blocks that, when assembled correctly, deliver exceptional properties. These materials exhibit a highly complex hierarchical structure, spanning from the nano- to the micro- and macro-scale, enabling them to achieve superior properties that are often incompatible in conventional materials, such as high strength combined with high toughness, as in the case of seashells [83].

To overcome these challenges and explore novel pathways in materials design, scientists drew inspiration from nature [84]. By studying the structures, functions, and processes found in biological systems, researchers can mimic and adapt these principles to create innovative materials with improved properties. 

For instance, the self-cleaning abilities observed in lotus leaves [85], the remarkable strength and flexibility of spider silk [86], the adhesive capabilities of geckos’ fingertips [87], and the anisotropic compression response of honeycombs from bees’ hives [88] have inspired researchers in their quest to develop novel materials. Recent advancements in the field have focused on investigating and replicating auxetic materials, which possess a negative Poisson’s ratio [89], as well as lightweight cellular solids [90,91]. 

By drawing inspiration from nature’s principles and structures, researchers are paving the way for the development of advanced materials that possess exceptional properties overcoming existing limitations in conventional materials [92,93,94]. Such materials not only offer potential improvements in various industries but also provide insights into the fundamental understanding of materials science. The fascinating intersection of bioinspired materials design with innovative technologies, such as AI, enhances the efficiency and effectiveness of design processes. By connecting the intricate geometries found in biological materials and taking advantage of AI algorithms, researchers can unlock new frontiers in materials science, leading to the development of advanced materials with unprecedented properties and functionalities.

To date, the study and design of bioinspired structures have relied on an empirical, extensive, and time-consuming top-down strategies without the aid of AI algorithms. Scientists engage in careful observation and analysis of natural organisms to identify the fundamental principles and structures that contribute to their remarkable properties [95,96,97,98]. This involves examining the arrangement of building blocks, hierarchical organization, and interactions at various scales within the materials. Researchers employ qualitative and semi-quantitative analysis techniques, such as microscopy, spectroscopy, and computational modeling, to gain a comprehensive understanding of the structural and chemical composition of these biological systems. Once these nature-inspired designs are well understood, researchers can replicate and adapt these principles to tune synthetic materials with similar properties. Such an iterative process involves designing and fabricating materials using advanced manufacturing techniques, conducting experiments to validate their performance, and refining the designs based on the outcomes. However, this approach is resource-intensive and time-consuming, often requiring substantial trial and error.

To overcome these limitations and expedite the bioinspired materials design process, the integration of AI algorithms can greatly enhance the efficiency and effectiveness of the approach. Indeed, the arrival of the AI-based bioinspired materials design provides several advantages that streamline researchers’ work. By using supervised AI (see Section 2.1), researchers can accelerate materials development by quickly exploring a vast design space, decreasing the pool of potential solutions, and identifying optimal material compositions and structures for the chosen application [99]. However, supervised learning requires high-quality data with accurate labeling about behaviors and characteristics to work correctly. This can be done only by accessing an extensive dataset of natural resources in which the information can be listed as images of materials, to which must be attributed known behaviors and characteristics. Unfortunately, supervised learning needs high quality data with accurate labeling to work correctly, which is not always possible in case of designing completely new materials. For this reason, efforts are underway to construct large and well-established material databases, such as the Materials Project [100], MatWeb [100], or PolyInfo [100], which provide usable information.

**Figure 2:**
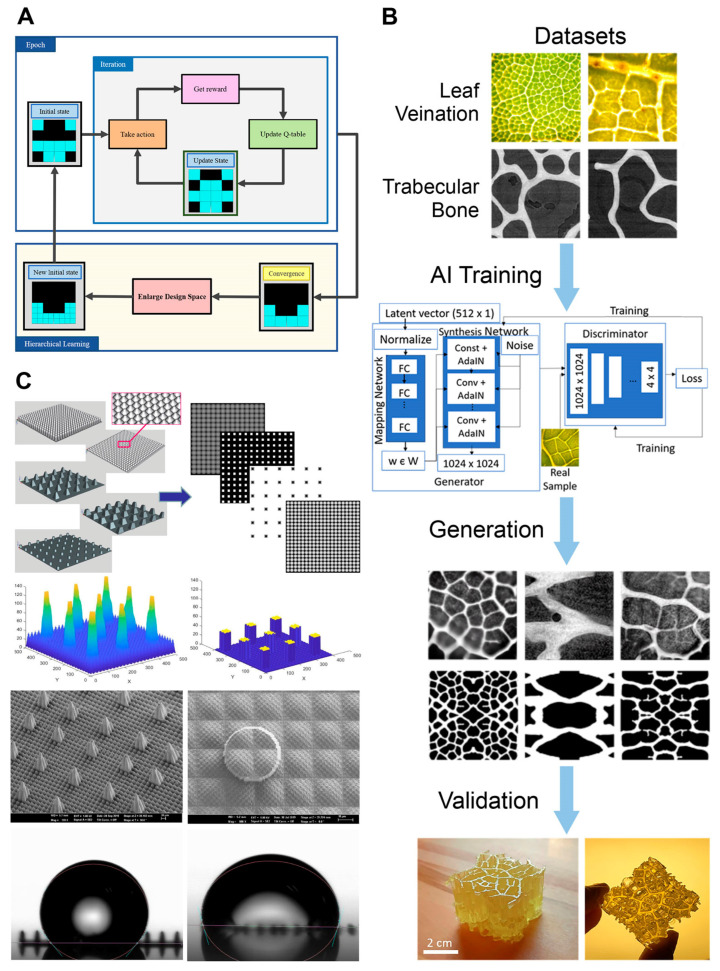
(**A**) Enhanced hierarchical design approach that accelerates the search speed for more effective results by expanding the exploration set upon convergence. Reproduced with permission from Ref. [101]. CC BY 4.0. (**B**) AI generation of bioinspired structures. Starting from leaf and bone datasets, GAN, after a training phase, is capable of generating leaf-like (**left**), bone-like (**center**), and hybrid leaf/bone (**right**) images, providing novel architectures at different hierarchy levels. The proposed structures were 3D printed for subsequent validation of mechanical properties. Reproduced with permission from Ref. [56]. CC BY 4.0 (**C**) AI-analyzed microtextured surfaces with low and high degrees of wettability. The dataset was constituted by Matlab models from a grayscale height map and converted into a colored height map (**up**), SEM images and empirical wettability tests were employed to verify the ANN prevision (**down**). Reproduced with permission from Ref. [102]. CC BY.

On the other hand, because the datasets are not complete enough to offer fully reliable results, researchers have explored the usage of alternative AI models. For instance, Yu et al. [101] utilized reinforcement learning to achieve a tuned design from an unknown design space, obtaining a model that can learn a biological design strategy through multiple iterations of training. In their approach, they provided a finite element method (FEM) that calculated mechanical properties as “reward values” for the algorithm in order to obtain materials with high fracture toughness. To ensure highly optimized solutions and increase confidence, the AI initially analyzed a small set of systems. After, the size was gradually increased each time the convergence of results was achieved. This approach aimed to reduce the calculation time for obtaining the best design. During the training process, the model is capable of learning the biological design strategy, which allows it to be extended to more complex structural optimization problems by providing different types of mechanical properties as reward values (Figure 2A). Modifying the system’s intricacy or incorporating additional variables as rewards, this innovative design framework finds versatile utility across various applications. For example, it can involve the utilization of over three diverse materials in the construction of a 3D structure, while simultaneously factoring in dual attributes such as energy absorption and strength to assess structural integrity. Hence, it is strongly conceivable that this design approach holds the potential to address a multitude of intricate structural design challenges, thereby showcasing its wide-ranging relevance within the realm of bioinspired materials engineering. Table 3 summarizes the ML models for each cited article and the related issues addressed in the field of materials design.

Another example is constituted by the work of Shen et al. [56], where an unsupervised deep learning model called a generative adversarial network (GAN) was used to generate bioinspired 2D and 3D structures from a series of natural images. The training process involved pairing two internal neural networks, a generator and a discriminator. While the first generated convincing sample images the latter learned to distinguish real data from artificial data. By starting with a leaf dataset, the GAN was able to generate images with scale-specific feature control, resulting in hierarchical structures similar to those found in nature. Although the artificial microstructures had an effective modulus that surpassed all the training structures, the generated structural families were limited to three due to the limits of the leaf dataset. However, when the leaf and bone datasets were mixed, the GAN produced unique microstructures with varying degrees of hierarchy. The flexibility of the algorithm allows for its application to other material classes. However, it is important to note that the results obtained from GAN simulations need to be validated. Indeed, a combination of computational simulations and experimental validation is necessary to verify the effectiveness and reliability of the generated structures before they can be applied in practical settings (Figure 2B). Overall, this study demonstrates how the generative GAN algorithm could effectively leverage inherent patterns within natural materials, exemplified by the leaf dataset, to facilitate the design of engineered materials. The method’s potential lies in its versatility, as it can be extended to a wide array of material categories, including auxetic structures. This extension can be achieved by augmenting the dataset with pertinent material properties beyond just the modulus—properties such as the yield strength, toughness, or Poisson’s ratio. These supplementary properties could be derived from simulated stress–strain curves or obtained by configuring simulations to provide additional insights. Furthermore, adapting the optimization criterion through which the genetic algorithm navigates the latent space opens up further avenues for exploration. However, when transitioning to practical applications, it’s important to consider scenarios where empirical data harmonizes with simulated results. Achieving this harmonization might necessitate normalizing simulations using experimental data or validating simulations against it.

By using materiomics coupled with physics-based deep learning [103], AI algorithms can predict complex behaviors and discover new material compositions and architectures, leading to enhanced properties. For instance, Ding et al. [104] developed a novel long short-term memory (LSTM) approach to design a bioinspired structure with simultaneous optimal stiffness and toughness. The boundary conditions were set considering mechanical parameters such as the Young’s modulus, failure strain, and Poisson’s ratio for the final involved materials. After a previous training phase, in the time of five to ten iterations it is possible to obtain the optimal sample configuration for the desired properties.

Differently, Lantada et al. [102] employed artificial neural networks (ANNs) to predict the wettability of biological and bioinspired structures based on their hierarchical surface features. The authors created a comprehensive library of bio-surfaces with well-known wettability properties, which served as training data for the ANN model. Subsequently, new unique topographies were introduced to evaluate the performances of the algorithm. The physical structures were manufactured, and the wettability results were compared with those predicted by the AI model. The convergence of results highlighted the remarkable capability of AI assistance in identifying novel bio-interfaces with hierarchical tribology, enabling precise control over wettability. This approach could consider a wide array of variables delivering many regulated features and behaviors, thus providing greater adaptability to specific applications (Figure 2C).

Lastly, the use of AI can decrease the costs associated with materials development. Indeed, by streamlining the design process, the need for extensive laboratory experimentation is reduced. As evidence, Gu et al. [8] calculated the mechanical properties of 100,000 natural microstructures in terms of computational cost. Using FEM, the process took around 5 days, while using their developed ML approach, the training process took from 30 s to 10 h and the predictive phase took less than a minute to solve for the same amount of data. 

In such a way, by leveraging AI’s computational power, scientists can focus on the most promising solutions and accelerate the materials design process, making it more efficient and cost-effective.

Overall, AI’s capabilities in data analysis, pattern recognition, and predictive modeling empower researchers to harness the potential of bioinspired materials more effectively, pushing the boundaries of materials science and engineering.

**Table 3:** Common issues addressed using ML and AI in materials design.

Biologically Inspired Materials		
ML Model/AI Algorithm	Common Issues Covered	References
Reinforcement learning (RL)	Composite materials structural optimization	[101]
Generative adversarial network (GAN)	Generation of bioinspired structures from images	[56]
Long short-term memory (LSTM)	Generation of bioinspired structures from fixed mechanical properties	[104]
Artificial neural network (ANN)	Prediction of wettability from bio-surfaces	[102]
Convolutional neural network (CNN)	Cutting of computational costs for calculation of bioinspired structure properties	[8]
Mechanical materials		
Gradient boost machine with least absolute shrinkage and selection operator (GBM-LASSO)	Young’s modulus prediction	[105]
Random forest (RF)	Microscale elastic strain field prediction	[106]
GAN	Microstructure and mechanical properties relation of composite materials	[37]
ANN and linear regression (LIR)	Prediction of mechanical behavior from microstructures	[107]
Accumulative roll bonding (ARB)	Generation of composition and tensile properties prediction	[108]
ANN	Prediction of non-linear structural deformations	[109]
Recurrent neural network (RNN)	Prediction of material plasticity	[110]
Convolutional LSTM	Prediction of fracture behavior	[49,111]
ANN	Prediction of tensile strength of nanocomposites	[112]
ANN	Prediction of flexural modulus and bending shear of nanocomposites	[113]
Compositionally complex materials, soft matter, and metamaterials		
Hybrid active-learning	Design of high entropy alloys	[114]
RF	Prediction of thermodynamic and composition for generation of ceramic materials	[115]
Reinforced learning for structural evolution (ReLeaSE)	Design of de novo molecules	[116]
ANN	Prediction complex systems behavior	[117]
Gaussian process regression (GPR)	Prediction of tensile strength of polymer–CNT composites	[118]
ANN	Prediction of thermal properties of conductive nanocomposites	[119]
Integrated deep neural network (DNN)	Prediction of stress–strain responses of porous metamaterials	[120]
CNN	Optimal structural design of 2D metamaterials	[121]

## 4. AI Algorithms for Mechanical Prediction

The exploration of mechanical properties plays a pivotal role in comprehending material behavior. By delving into this field, it is possible to gain valuable insights not only for the creation of novel materials but also for enhancing existing ones [122,123,124]. Nevertheless, predicting the structure and properties of materials has perpetually posed a challenge in material science. Traditionally, the development process involves investigating and fine-tuning numerous parameters individually, subsequently combining them to estimate the characteristics of novel materials. However, simple simulations fall short in providing quantifiable values for all mechanical properties. Consequently, a trial-and-error approach becomes necessary to unravel the correlation between design parameters and mechanical behavior [125,126].

The integration of AI into mechanical materials presents an innovative approach that revolutionizes the predictive modeling process. By harnessing the capabilities of AI, particularly in terms of ML algorithms such as LIR and SVR, it becomes feasible to accurately identify intricate relationships among a wide array of variables. This breakthrough enables the efficient and cost-effective prediction of mechanical properties, ultimately simplifying the design of advanced engineering materials [7,127,128]. In this context, to predict materials properties, AI undergoes a training phase on a dataset, followed by the implementation of a predictive process to estimate particular properties. Generally, a predictive model is accompanied by a validation or testing procedure to assess the performances of the algorithm.

The tensile properties of a material play a crucial role in determining its overall performance, durability, and suitability for specific applications. Thus, their accurate prediction has great importance for their usability. Young’s modulus, or the elastic modulus, is one of the key parameters that identify the ability of a material to withstand changes in length under load, measuring its resistance to stretches and deformations. Young’s modulus is strictly related to the stress and strain values experienced during tensile tests and is influenced by numerous factors, such as the chemical and physical structure of the material. These elements include the nature and arrangement of interatomic bonds, the presence of impurities, pore diffusion and size, and the environmental conditions during employment [129]. Hu et al. [105] utilized ML techniques to predict the Young’s modulus of SiO_2_-based glasses. The training dataset was generated by high-throughput molecular dynamic (MD) simulations completed by the presence of a set of descriptors that generalize the main chemical relations of SiO_2_-based glasses. The predictions of the ML model were then compared and validated with a large amount of both simulation and experimental data, revealing its outstanding prediction capability compared to other frequently used ML models. An impressive aspect of the model is its ability to extend its applicability to involve new types of oxides by incorporating a small amount of relevant MD data into the training set. This highlights the model’s versatility and potential for further advancements in predicting material properties.

**Figure 3:**
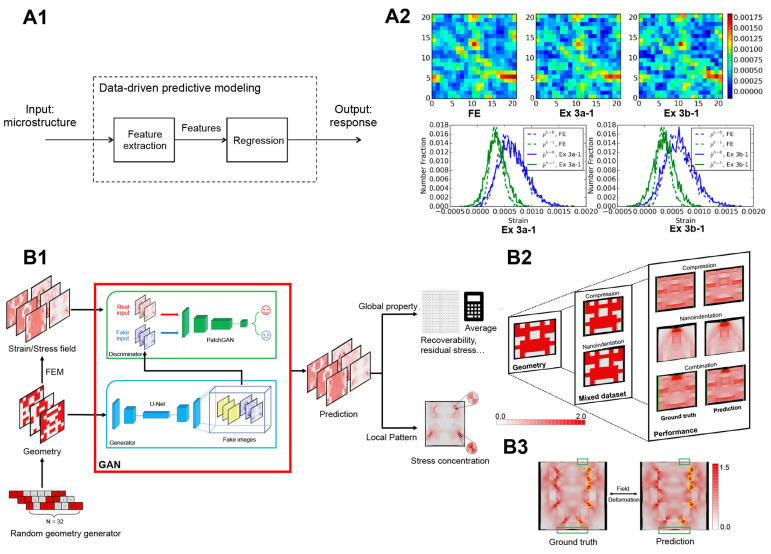
(**A1**) Schematic representation of the data-driven predictive modeling process, which involves two main steps: feature extraction and the construction of a regression model. (**A2**) Comparison between FE-simulated and refined strain field predictions (from two different regression models). The upper section displays color maps representing the strain fields, while the lower section presents strain distribution plots. Reproduced with permission from Ref. [106]. CC BY 4.0. (**B1**) GAN-based workflow for predicting the mechanical behavior of composite materials based on their microstructure. The process involves utilizing a generator, which generates synthetic images with inaccurate fields (blue window), and a discriminator, which compares these fake images with real images obtained from FEM analysis (green window). (**B2**) The model can accurately identify the loading conditions and predict stress fields under compression, nanoindentation, or a combination of both settings. (**B3**) The field predictions are validated using FEM models, and a direct comparison of the deformation and stress fields is depicted using green and yellow lines, respectively. Reproduced with permission from Ref. [37]. CC BY 4.0.

The ability to reliably predict stress and strain fields with the assistance of AI would greatly benefit researchers, saving them valuable time by reducing the need for numerous mechanical tests. Recognizing this advantage, Liu et al. [106] investigated advanced concepts in ML and data mining focusing on feature extraction, feature ranking and selection, and regression modeling techniques for more efficient predictions of microscale elastic strain fields in a three-dimensional voxel-based microstructure volume element (Figure 3A1) compared with FEM analysis. The authors discovered that ensemble methods, which combine multiple weak regressors concentrating on different subdomains of the original task, hold great potential. By combining basic feature descriptors with engineered features, the ensemble methods significantly enhanced prediction performance. Additionally, they found that utilizing a reduced set of descriptors generated by feature ranking methods produced even better results. In terms of regression techniques, ensemble methods such as random forests proved superior accuracy and reduced time consumption (Figure 3A2).

Furthermore, Yang et al. [37] reported the use of a GAN to establish a connection between the microstructure and the mechanical behavior of composite materials. Through the production of geometrical images using a random generator, the authors performed FEM analysis to extract stress and strain fields during simulated mechanical tests. Despite the relatively limited dataset, the training of the DL model to distinguish real images containing FEM-calculated fields from fake images achieved remarkable accuracy. The GAN successfully predicted physical fields directly from the microstructure geometry of the material, exhibiting exceptional performance not only in the function for which it was designed but also in estimating derivative material properties. Moreover, enabling predictions of complex material behavior without constraints such as shape, boundary conditions, or geometrical hierarchy can extend the proposed approach. This method provides significant potential for performing physical modeling and simulations, presenting a significant advancement in efficiently evaluating the mechanical properties of hierarchical materials considering their geometrical structure only (Figure 3B).

Yield strength, ultimate tensile strength, and yield ratio are other significant tensile properties extensively investigated to evaluate material capabilities. Jung et al. [107] developed a DL algorithm utilizing ANN and LIR to predict the behavior of high-strength steels based on the volume fraction of their microstructure. Although the ANN algorithm failed to unveil the direct physical relationship between microstructural volume fraction and mechanical properties, it effectively discerned patterns within the experimental data. As a result, it successfully predicted important metrics such as yield strength, tensile strength, and elongation. Notably, the model demonstrated remarkable accuracy in predicting experimental data. This outcome highlights the efficacy of the DL algorithm in providing reliable predictions for high-strength steel properties, bringing microstructural information as a valuable input. Moreover, Najjar et al. [108] employed an ML method, called accumulative roll bonding (ARB), to formulate the composition and then predict the tensile properties of aluminum nanocomposites reinforced with SiC particles. After a first training phase, ARB investigated the distribution of particles and their impact on the composite material. Remarkably, the application of the ARB approach resulted in significant improvements in the ultimate tensile strength, yield strength, and hardness of the nanocomposite. The developed ML model’s efficiency was evaluated against other methods, considering performance criteria. Encouragingly, the model exhibited high accuracy and confidence in predicting the tensile yield and ultimate strength, as well as the elongation of all the produced composites, with an R^2^ value of 0.98. These findings demonstrate how ML methodologies can bring advancements in materials design, promoting a significant enhancement in material performance while reducing costs.

Non-linear mechanical problems pose the greatest challenges and computational demands in materials research. These issues encompass areas such as plasticity estimation, fracture mechanics, and dynamic stiffness assessment. To address the challenges in predicting non-linear structural deformations of materials, Stoffel et al. [109] undertook a study utilizing an ANN intended to overcome the obstacles associated with accurately forecasting such deformations. Initially, they employed experimental data to model the complete structural response. However, when attempting to predict additional deformations beyond the trained data, discrepancies occurred between the predicted and measured mid-point deflections of the samples. To overcome this challenge, an intermediate step was introduced to ensure that the state variables encountered in the intelligent FEA fell within the provided set of constitutive training data. By adopting this approach, the simulation results for the non-linear stress–strain field exhibited significantly improved accuracy compared to the initial method, and the computational effort required was considerably reduced compared to traditional FEA simulations. This advancement offers a promising alternative for researchers seeking precise predictions of material behavior while optimizing computational resources. The ANN-based approach proposed by Stoffel and colleagues could be employed to predict deformation behavior and mechanical properties of different metal alloys under various loading conditions. This could aid in optimizing alloy compositions for desired performance characteristics. Furthermore, such methods can be adapted to predict the deformation responses of polymers and biomaterials, helping in designing materials with tailored mechanical properties for specific interaction with biological systems under different conditions.

**Figure 4:**
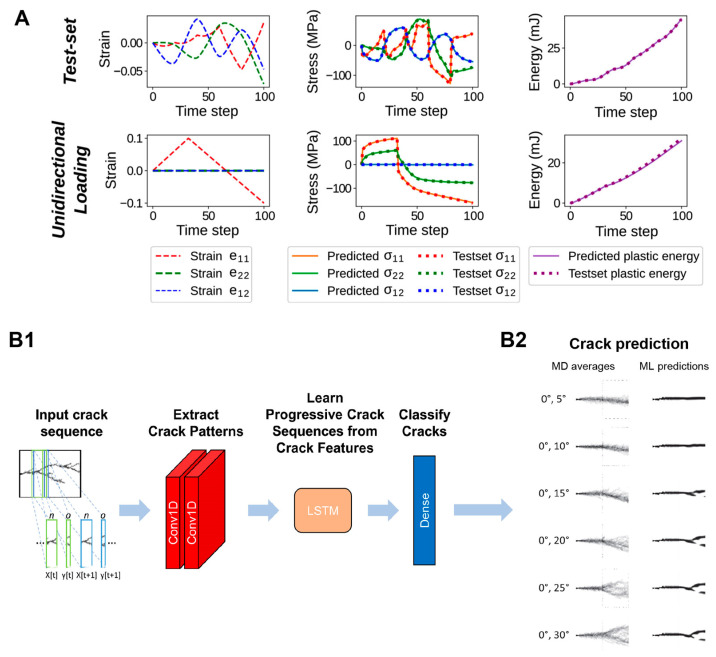
(**A**) Comparison of results from two representative volume elements (RVEs) subjected to distinct loading conditions. The first column displays the applied average strains, the second column depicts the average stresses predicted by the RNN model (solid line) in comparison to FEM (dashed line), and the third column illustrates the plastic energies predicted by the RNN (solid line) and FEM (dashed line). Reproduced with permission from Ref. [110]. CC BY 4.0. (**B1**) ML model containing two convolutional layers to learn geometric features of cracks, namely, an LSTM layer to learn relations and a dense layer to classify the results. (**B2**) The model can reasonably predict crack nucleation even from complex graphene systems. Reproduced with permission from Ref. [111]. CC BY 4.0.

In a different study, Mozaffar et al. [110] demonstrated the precise and efficient prediction of material plasticity using an RNN model. Even in this case, the approach eliminates the need for iterative empirical tests. The training datasets consisted of representative loading computer simulations incorporating constituents and microstructures, enabling the model to directly learn reversible, irreversible, and history-dependent phenomena from data. The RNN algorithm was trained using non-temporal microstructure descriptors from a comprehensive database comprising 8000 samples, with 80% allocated for training purposes. To evaluate the quality of results, validation tests were conducted. Remarkably, the outcomes from linear unidirectional loading tests revealed the ability to determine plasticity-constitutive laws for general materials and material classes with unprecedented accuracy and efficiency. Even complex phenomena such as distortional hardening could be predicted with an error less than 0.5%. The methodology’s flexibility and its direct relevance to materials design highlight its usefulness across numerous fields. Therefore, the approach is versatile and can be applied to different types of time-dependent and history-dependent data. Consequently, the combination of high-fidelity and high-throughput computational predictions of material behavior, along with the evolving field of AI assisted research, will undoubtedly pave the way for discovering new materials and designing structures capable of surpassing elastic limits and achieving exceptional performance levels (Figure 4A).

In the drive to design engineered materials, it is crucial not only to optimize their mechanical performances but also to ensure their durability over time. Fracture mechanics poses a significant challenge in material design, primarily due to the limitations of traditional prediction methods, which often require more accuracy. To address this issue, Lew et al. [111] set an ML approach as a powerful tool for understanding and predicting precisely this complex physical phenomenon in 2D materials, specifically focusing on graphene. The ML model was utilized to investigate the parameters required to calibrate predictions concerning fracture behavior based on crystal orientation. The ML model consisted of multiple layers, including two convolutional layers responsible for capturing geometric features of crack slices, an LSTM layer to learn sequential relationships among them, and a dense layer for result classification. The outcomes of the study demonstrated the remarkable capability of the DL model to quantitatively capture graphene fracture behavior. The predicted results were closely aligned with the existing literature’s findings, even when considering scenarios involving surface conditions, point defects, and grain boundaries that varied significantly from the original training data. The study emphasizes the possibility for exploring other materials, as the ML approach can be expanded to handle cases of increasing scale and complexity that are often too impractical for traditional MD simulations to directly address. (Figure 4B). Similarly, Hsu et al. [49] succeeded in the application of a ML model to predict the overall crack propagation pathways on bi-crystal materials (not included in the training dataset).

The pursuit of advanced materials tailored for technological progress has also steered the field of material science towards a keen focus on nanomaterials. The integration of nanotechnologies into various material matrices has undergone rapid evolution, imparting transformative mechanical properties hinging on nanomaterial attributes such as size, shape, concentration, and composition. As nanoscale challenges unfold, often involving the nuances of quantum physics due to the diminutive particle dimensions (ranging from 1 to 100 nm), a necessity arises for precise computational simulations to unravel and foresee the intricate inner interactions and properties of nanocomposites.

However, the computational route proves to be resource-intensive and time-demanding, prompting researchers to harness the potential of AI and ML tools for more expedient and cost-efficient explorations into the structure-property relationships of materials [130].

To this end, Harsha and colleagues [112] employed an ANN to prognosticate the tensile strength of Al/Al_2_O_3_ nanocomposites. By feeding the ANN with inputs including the quantity of Al_2_O_3_ nanoparticles, the hardness, and the elongation percentage, the AI algorithm adeptly gauged the strength, aligning it with the weight percentage of reinforcement. Notably, an augmentation in the neuron count within the hidden layers substantially heightened the prediction prowess, yielding an impressive 98% accuracy.

In a parallel endeavor, Nawafleh et al. [113] developed a bespoke ANN system tailored to foresee the mechanical traits of 3D printed thermoset epoxy/carbon nanofiber composites, even under the constraints of limited experimental datasets. This specialized ANN system employed three distinct model techniques to prognosticate the bending stress and flexural modulus of the composites, its architectural design facilitating precise predictions with a minimal number of experimental tests. This innovative approach empowered the optimization of fiber content, thus achieving the desired comprehensive mechanical performance for the final composite material.

Overall, the application of AI-based prediction methods for mechanical properties in material design and discovery holds paramount importance. These advanced techniques enable researchers to overcome the limitations of traditional empirical approaches and computationally expensive simulations. By harnessing the power of ML and DL algorithms, accurate predictions can be made regarding the mechanical behavior of materials under various conditions, facilitating the identification of novel materials with desired properties. This not only accelerates the materials discovery process but also provides valuable insights into the underlying physics and chemistry governing material behavior. With the fusion of ML algorithms and MI tools, researchers can navigate the vast design space efficiently, leading to the discovery of innovative materials that exhibit exceptional mechanical properties, durability, and functionality approaching and overtaking the traditional modeling methods.

## 5. AI Algorithms in Complex Materials, Soft Matter, and Metamaterials

In the field of compositionally complex materials, researchers face an increasing number of hurdles when it comes to designing, modeling, and predicting the properties of such materials. This challenge stems from their intricate nature, which exhibits a range of dynamic components. These components encompass internal structural interactions marked by competing kinetic and thermodynamic phases, significantly influencing the ultimate properties of the material. To effectively use such advanced materials, it becomes imperative to understand the intricacies of this non-equilibrium state between constituent elements and their environment [131].

Similarly, the exploration and development of soft materials struggles with analogous constraints. However, the level of complexity reaches unprecedented levels due to the presence of amorphous or disordered molecular arrangements that define these materials. This chaotic molecular state endows soft materials with a heightened sensitivity to even minor perturbations, primarily because of the significant impact of weak non-covalent interactions that strongly influence their ultimate properties [132].

In both scenarios, the emergence of AI rises as an essential ally. In particular, hybrid AI methods present remarkable opportunities for modeling and predicting properties of both compositionally complex materials and soft materials. These approaches effectively tackle intricate non-linear physics-related challenges, such as multiphysics problems, sparse data limitations, and the integration of atomistic and continuous simulations [131]. Additionally, they are proficient in predicting chemical defects or surface mutations, thereby facilitating enhanced discoveries regarding the relationship between structure, chemistry, and material properties.

Nevertheless, the current integration of AI algorithms with existing dynamic simulations (e.g., molecular dynamics, kinetic Monte Carlo methods, and phase-field theory) has its constraints, limiting the scope of exploration to simpler functional forms when compared to the capabilities of ML. As a result, this approach may overlook the intricate mathematical relationships underlying the correlations between composition, structure, and material properties in chemically complex materials. The existence of numerous non-linearities, complex interactions, microstructure features, phase states, and a high degree of chemical dimensionality implies that leveraging the benefits of traditional correlative AI, without imposing arbitrary “physics-based” restrictions, may offer a more comprehensive understanding of these challenges [131].

For instance, Rao et al. [114] recently reported a fully integrated hybrid active-learning AI model to accelerate the design of novel high-entropy Invar alloys. Their approach works as a closed-loop workflow, integrating ML with density-functional theory simulations, thermodynamic calculations, and experiments. The authors emphasized the proficiency of this method in designing high-entropy Invar alloys, even with limited experimental data. Notably, the entire workflow was completed within a few months, in contrast to the conventional alloy design approach that typically spans years and requires a significantly larger number of experiments. Based on these findings it is possible to believe that this method could be a suitable pathway for the fast and automated discovery of high-entropy alloys with optimal thermal, magnetic, and electrical properties.

Kaufmann et al. [115] proposed an RF approach blended with density functional theory for the identification of thermodynamic and compositional features of multi-component-disordered metal carbides. Using this hybrid approach, 70 new ceramic materials were identified and predicted, some of which were then validated.

When considering soft matter design, it is essential to recall that estimating final behavior is necessary to correlate the macroscopic environment, mesoscale morphology, and nanoscale chemical composition and physical arrangement. Indeed, due to the complexity of soft materials, intuition-based design is a difficult path to approach. For this reason, ML models integrated with MI can provide deeper physical insights compared to the pure physics-based modeling methods. In this context, coupling modeling techniques, such as the smooth overlap of atomic positions technique, with an ML algorithm gives scientists the ability to characterize the properties of soft materials. Given the presence of both disorder and heterogeneity in such a system, it is possible to obtain, for instance, the relative energies of organic crystals or intermolecular electronic couplings. Therefore, generative design methods for both molecular and morphological structure discovery are of great interest to the soft matter community [132]. For instance, the variational autoencoder (VAE) framework [133] should provide useful insights for the generative design of soft matter chemistry and morphology from a multidimensional continuous representation of the latent space of molecules [134]. Similarly, deep reinforcement learning can be used to discover novel soft compounds optimized for a single property or multiple desired properties. Using this approach, Popova et al. [116] introduced a pioneering computational strategy, known as reinforcement learning for structural evolution (ReLeaSE), for the de novo design of molecules with desired properties. This innovative approach was successfully employed to design chemical libraries exhibiting a preference for structural complexity or compounds with specific physical properties, including maximal, minimal, or a specific ranges of traits such as melting point or hydrophobicity. Additionally, the ReLeaSE method was effectively utilized to target compounds with inhibitory activity against Janus protein kinase 2, showcasing its versatility in various molecular design applications.

A noteworthy technique in soft matter research is coarse-grained (CG) modeling. With ML demonstrating remarkable success in electronic structure prediction and atomistic simulations, its application in CG simulation has emerged as an intriguing tool for researchers. In this context, Zhang et al. [117] introduced an ANN that produces CG potential models without relying on approximations, aligning closely with ab initio simulations. Traditional methods struggle to discover and manage a vast number of defined collective variables, making it nearly impossible to tackle complex system behavior. However, ML techniques offer a more streamlined design process by effectively handling this challenge.

Additionally, anticipating the behavior and attributes of composite materials born from the union of soft materials with nanoscale counterparts stands as a captivating and front-line domain that intersects materials science and nanotechnology. An illustrative case involves the amalgamation of elastomers with nanoscale reinforcements such as carbon nanotubes (CNTs), revered for their impressive mechanical prowess, elongated aspect ratio, and distinctive electrical characteristics. This synergistic blend engenders a composite of unprecedented and amplified attributes that elude either constituent material in isolation, rendering it exceptionally versatile and applicable across a diverse spectrum of uses [135].

However, the interplay of numerous factors at macro, micro, and nanoscale tiers ushers in a profound complexity, challenging traditional computational methodologies. Unfortunately, this intricacy mandates laborious and resource-intensive experimental approaches to validate predictions and optimize the performance of these soft nanocomposites [130,135]. To streamline the design trajectory for these materials, Le harnessed a Gaussian process regression (GPR) model built on ML principles, foreseeing the tensile strength of polymer–CNT composites [118]. This adept model incorporated a staggering eleven input variables spanning polymer and CNT properties, encompassing factors such as matrix type, CNT weight fraction, and CNT modification technique, among others. Impressively, the GPR model showcased robust performance, boasting minimal root mean squared and mean absolute errors. This prowess empowers the machine learning technique to predict the behavior of CNT–polymer nanocomposites at the outset of design, circumventing the need for initial physical experiments.

The integration of nanoscale materials into soft polymeric matrices not only reshapes mechanical attributes but also triggers a cascade of assorted features, reshaping the final nanocomposites’ conduct. This phenomenon comes to the fore in conductive filler-infused polymeric composites, sought after across a multitude of industries for their pliability and heightened electrical conductivity. However, these nanocomposites grapple with a quandary, as their elevated electrical conductivity can yield Joule heating and consequent heat generation. To tackle this challenge, Kil et al. [119] developed a pair of ANN models that were sculpted to foretell the self-heating traits of polydimethylsiloxane composites housing CNTs and CF. The predictive prowess of these models was gauged against experimental values to validate their efficacy. The data-driven predictive models accurately gauged surface temperature and electrical resistance, yielding R-squared values of 0.91 and 0.97, respectively. This underscores the potency of ANN models in optimizing nanomaterial concentrations within flexible polymeric matrices and predicting self-heating quandaries in conductive nanocomposites.

As a matter of fact, when properly applied, these methods can enable the efficient identification and characterization of promising novel materials and nanomaterials. However, the intrinsically disordered nature of soft (nano)materials requires simulation over hierarchically organized material scales, as well as effective descriptions of the complex many-body interactions underpinning phenomena, which have slowed progress compared to other material classes [132].

Lastly, a captivating materials class is constituted by metamaterials distinguished by their anthropic design and unique features absent in nature (such as a negative Poisson ratios or refractive indexes) [136,137]. These characteristics arise not only from their molecular composition and arrangement but also from their complex geometric structures. The mechanical properties, shear resistance, and absorption of electromagnetic or sound waves of these materials are deeply influenced by their shape, offering benefits for a wide spectrum of applications [138,139]. The complex and unconventional behavior exhibited by metamaterials makes them ideal candidates for investigation using AI techniques.

Buehler [120] presented a DL strategy that enables the prediction of stress–strain responses in highly porous metamaterials with hierarchical microstructures. This DL strategy not only captures the relationship between microstructure and mechanical behavior but also faces the inverse design problem. By specifying a desired stress–strain response, the model can generate a de novo specific microstructure to achieve the desired outcome. This innovative framework exemplifies the transformative potential of AI in advancing the understanding and development of metamaterials, opening up possibilities for the discovery of new hierarchical metamaterials.

**Figure 5:**
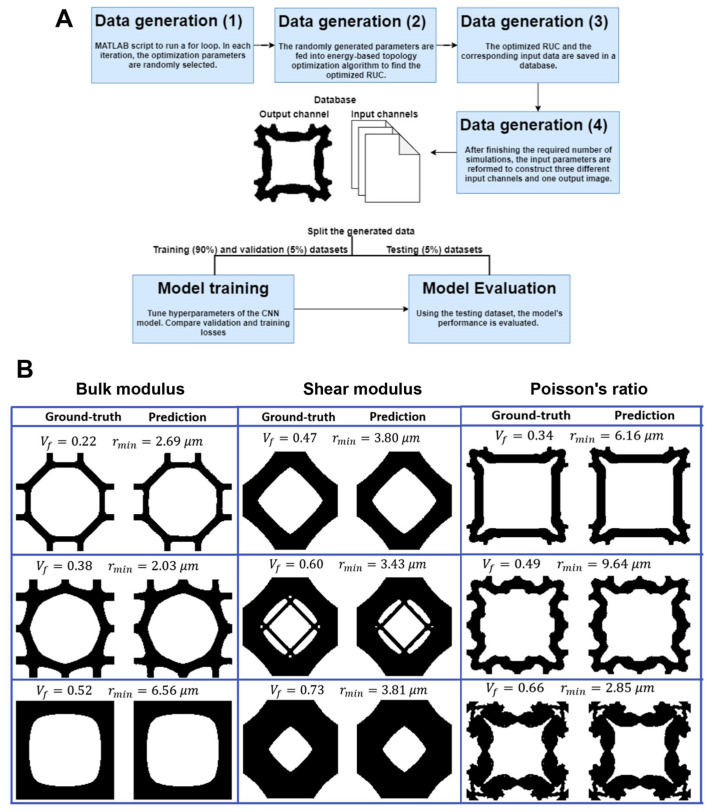
(**A**) Illustrative diagram depicting the stages of CNN development with an integrated optimization process. (**B**) Comparison between optimized designs for the three requirements: maximizing the bulk modulus, maximizing the shear modulus, and minimizing the Poisson’s ratio. Reproduced with permission from Ref. [121]. CC BY 4.0.

Kollmann et al. [121] developed a DL model based on CNN to predict optimal designs for metamaterials. Their study focused on three specific tasks: maximizing the bulk modulus, maximizing the shear modulus, and minimizing the Poisson’s ratio. The CNN architecture contained three key components: the encoder, which compresses input images into compact representation by a series of convolutional layers; the decoder, which receives the encoded inputs and constructs an image by mapping the targets to represent the unit cell; and the bridge, which links the encoder and the decoder, facilitating the flow of information. To generate the necessary data for training the model, a topology optimization framework was utilized, resulting in three input channels and one output image. During the training phase, energy-based homogenization methods and periodic boundary conditions were employed to evaluate effective properties and define a well-posed optimization problem. The proposed 2D microstructures were discovered non-iteratively and nearly instantaneously for various input parameters. The developed DL model exhibited robustness in terms of both accuracy and inference time. The validation phase demonstrated the DL model’s ability to process data efficiently, even on low-computing-capacity platforms such as laptops. This accomplishment brings a more accessible method for topology optimization, reducing time and costs but keeping high accuracy (Figure 5A,B).

Overall, the employment of ML models in advanced materials design presents immense potential in tackling the challenges faced by the field. It could open up avenues for advancements, ranging from the accurate prediction of complex material properties to the optimization of designs for diverse applications. However, it is important to recognize that certain technical limitations still need to be addressed, such as establishing causality, integrating intricate physics-based relationships, and effectively handling high-dimensional phase spaces. Nonetheless, with ongoing technological innovation, the application of AI in materials design has the capability to bring about substantial improvements and pave the way for groundbreaking discoveries.

## 6. Current Limitations and Future Perspectives

As discussed above, AI has the potential to usher in a new scientific paradigm by enhancing and streamlining the acquisition of knowledge about materials. It can mitigate or eliminate research bottlenecks, allowing for faster progress in materials design. However, to fully leverage the power of AI- and ML-based methods, it is crucial to generate accurate and reproducible data on a large scale. This necessitates adopting an open, disciplined, and collaborative environment that establishes “agreements” on data communication, ML models, and experimental protocols.

Undoubtedly, this presents a significant challenge for the future of the materials design field. However, overcoming this challenge promises to bring transformative advancements, propelling materials discovery to unprecedented levels of efficiency and effectiveness. It is worth noting that while AI models have traditionally been perceived as black boxes, recent advancements in eXplainable AI [140] will provide methods and processes to understand and trust the results and output of machine learning algorithms. This will enable researchers to characterize the accuracy, transparency, and correctness of AI models in the decision-making process.

Another existing gap is the sparse usage of AI-based multiscale modeling techniques. While AI methods demonstrate proficiency at individual scales, a deficiency in research efforts exists concerning the establishment of connections across various scales. This quandary bears substantial importance, as the properties of materials are intricately woven into their inherent microstructures. This parallels the concept that, much like the biomechanical conduct of cells dictates the holistic physiological functions of the human body, these microstructural attributes govern material traits. Thus, an imperative call arises to fortify these interconnections in order to fully unlock the advantageous potential of these pivotal methodologies within real-world applications.

Furthermore, an enduring hurdle within this field pertains to the overarching capacity for models to generalize, which characterizes the model’s ability to respond to unseen data. As the use of deep language models proliferates in the domain of materials science, a deliberate investigation into the generalization potential of these models within materials-specific tasks, such as forecasting the physical and chemical phenomena of materials, emerges as an imperative stride toward the future. The utility threshold for such learning approaches stands to undergo a substantial expansion if they manage to encapsulate broadly applicable principles in a manner that enables the extrapolation of these functional relationships toward fresh solutions that are distinct from the training dataset provided. This expansion goes beyond the mere unlocking of predictive prowess; it also encompasses the elucidation of connections in a manner that resonates with human comprehension.

Looking ahead, such models will show significant potential for various materials research in a wide variety of complex tasks, such as automated material information extraction, protein folding, molecular property prediction, mechanical behavior, fracture pattern investigation, and the design of architected metamaterials, thus helping finally to map the intricate relationship between “composition–structure–property–processing–application”. Furthermore, as AI algorithms and ML models become more and more prevalent in materials science, there will be a concerted effort to investigate their ability to generalize across diverse materials-related tasks. This will involve understanding the models’ capacity to predict and explain physical and chemical phenomena in materials beyond their training data. Successful generalization will mark a significant expansion of the AI utility threshold, allowing researchers to extrapolate functional relationships and discover novel solutions.

Ultimately, one noteworthy future transformation will arise from the establishment of collaborative ecosystems between researchers, industries, and institutions that will be crucial in harnessing the full potential of AI in materials design, thus fostering the exchange of data, models, and insights. This collaborative approach will transcend geographical boundaries and disciplinary limitations, enabling a collective effort to tackle complex materials challenges. As AI-driven collaborative materials research gains momentum, the development of specialized AI tools and platforms will become a focal point, offering a tailored solutions for various stages of materials design, from initial conceptualization to advanced simulations. Such AI-powered platforms will democratize access to cutting-edge technologies, allowing researchers with diverse expertise to contribute to materials discovery.

## 7. Conclusions

In conclusion, the field of materials design is undergoing a transformation with the integration of AI and ML techniques. These technologies offer valuable tools to tackle the inherent complexity of exploring vast design spaces and investigating new materials or boosting the existing one. By working in conjunction with human creativity and ingenuity, AI algorithms and ML methods can contribute to the discovery and refinement of novel materials for future technologies, revolutionizing our approach to materials design, which may have a broad application in biomedical, engineering, and mechanics research.

The advancements we have discussed, such as bioinspired materials, mechanical materials, and advanced materials, serve as proof of concept for this new approach. Additionally, we have provided an overview of the main ML models and MI tools used in the field, which accurately predict various material properties (e.g., mechanical behavior, structural topology optimization, material plasticity, and fracture behavior). This overview serves as a guide for readers to delve further into these models and tools as part of their research activities.

In summary, although we are still at the early stages of this journey, we are confident that AI will serve as an amazing research assistant for materials scientists rather than a competitor. This will significantly expand the horizons of the field, opening up new possibilities and accelerating advancements in materials design.

## Figures and Tables

**Table 1:** Machine learning algorithms used in materials design and optimization.

Machine Learning (ML) Models			
Type	ML Methods	Applications	References
Supervised Learning	Linear regression (LIR), polynomial regression (PR), support vector regression (SVR), random forest (RF), artificial neural network (ANN), feedforward neural network (FFNN), multilayer perceptron (MLP)	Mechanical prediction, structural optimization, strength or hardness prediction, prediction of plastic behaviors	[7,23,24,25,26,27,31,32,33,43,44,45]
Unsupervised Learning	Generative adversarial network (GAN)n Neural network, *k*-means, principal component analysis (PCA)	Structural topology optimization, prediction of modulus distribution, composite design, multi-scale modeling, architected material design	[37,39,46,47,48]
Reinforcement Learning	Graph neural network (GNN), Monte Carlo, functional approximation method	Structure prediction, crystal structure, metamaterials design, architected material design	[39,41,49,50]
